# KAI1 suppresses HIF-1α and VEGF expression by blocking CDCP1-enhanced Src activation in prostate cancer

**DOI:** 10.1186/1471-2407-12-81

**Published:** 2012-03-06

**Authors:** Jung-Jin Park, Yeung Bae Jin, Yoon-Jin Lee, Jae-Seon Lee, Yun-Sil Lee, Young-Gyu Ko, Minyoung Lee

**Affiliations:** 1 Division of Radiation Effect, Korea Institute of Radiological and Medical Sciences, Nowon-Ku, Seoul 139-706, Korea; 2 Division of Radiation cancer Research, Korea Institute of Radiological and Medical Sciences, Nowon-Ku, Seoul 139-706, Korea; 3 College of Pharmacy & Division of Life & Pharmaceutical Sciences, Ewha Womans University, 11-1 Daehyun-Dong, Seodaemun-Gu, Seoul 120-750, Korea; 4 College of Life Sciences and Biotechnology, Korea University, 1, 5-ka, Anam-dong, Sungbuk-gu, Seoul 136-701, Republic of Korea

## Abstract

**Background:**

KAI1 was initially identified as a metastasis-suppressor gene in prostate cancer. It is a member of the tetraspan transmembrane superfamily (TM4SF) of membrane glycoproteins. As part of a tetraspanin-enriched microdomain (TEM), KAI1 inhibits tumor metastasis by negative regulation of Src. However, the underlying regulatory mechanism has not yet been fully elucidated. CUB-domain-containing protein 1 (CDCP1), which was previously known as tetraspanin-interacting protein in TEM, promoted metastasis via enhancement of Src activity. To better understand how KAI1 is involved in the negative regulation of Src, we here examined the function of KAI1 in CDCP1-mediated Src kinase activation and the consequences of this process, focusing on HIF-1 α and VEGF expression.

**Methods:**

We used the human prostate cancer cell line PC3 which was devoid of KAI1 expression. Vector-transfected cells (PC3-GFP clone #8) and KAI1-expressing PC3 clones (PC3-KAI1 clone #5 and #6) were picked after stable transfection with KAI1 cDNA and selection in 800 *μ*g/ml G418. Protein levels were assessed by immunoblotting and VEGF reporter gene activity was measured by assaying luciferase activitiy. We followed tumor growth *in vivo *and immunohistochemistry was performed for detection of HIF-1, CDCP1, and VHL protein level.

**Results:**

We demonstrated that Hypoxia-inducible factor 1α (HIF-1α) and VEGF expression were significantly inhibited by restoration of KAI1 in PC3 cells. In response to KAI1 expression, CDCP1-enhanced Src activation was down-regulated and the level of von Hippel-Lindau (VHL) protein was significantly increased. In an *in vivo *xenograft model, KAI1 inhibited the expression of CDCP1 and HIF-1α.

**Conclusions:**

These novel observations may indicate that KAI1 exerts profound metastasis-suppressor activity in the tumor malignancy process via inhibition of CDCP1-mediated Src activation, followed by VHL-induced HIF-1α degradation and, ultimately, decreased VEGF expression.

## Background

More than 20 metastasis-suppressor genes encoding products with specific metastasis-suppressing functions have been discovered [1]. KAI1 (CD82) was first identified as a prostate cancer metastasis suppressor through genetic screening [2]. Studies have shown that the expression of KAI1 is actually down-regulated in most metastatic cancers [3]. Consistent with this, reduced KAI1 expression is associated with malignant progression of human prostate cancer [4]. KAI1 is a member of the tetraspan transmembrane superfamily (TM4SF) of type III membrane proteins, specifically of the tetraspanin subgroup. It is ubiquitously expressed, especially in spleen, thymus, prostate and colon, and interacts with a large number of proteins, including integrins, epidermal growth factor receptor (EGFR), and other tetraspanins. KAI1 plays a role in organizing associated proteins into functional signaling networks that influence the metastatic potential of cancer as part of a tetraspanin web [5].

Metastasis is the final step in the tumor progression processes and involves increased invasiveness, extravasation into secondary organs, and angiogenesis [6]. Because cancer progression and metastasis involves multiple steps with a high degree of complexity, it requires the contribution of a variety of molecules. Hypoxia, which is the environmental factor best known to induce cancer metastasis, up-regulates the expression of vascular endothelial growth factor (VEGF), which, in turn, induces the formation of tumor-feeding vessels [7]. It has been documented that the transcription factor, hypoxia-inducible factor 1 α (HIF-1α), is stabilized in the context of reduced oxygen availability and stimulates the expression of VEGF [8]. An important step in HIF-1α induction is closely related to the activity of the tumor suppressor, von Hippel-Lindau (VHL) protein. VHL is viewed primarily as an ubiquitin ligase for HIF-1α that promotes the proteasome-dependent proteolytic degradation of HIF-1α [9]. Generally, a loss of VHL expression in cancer leads to up-regulation of HIF-1α and increased VEGF expression [10].

A previous proteomic analysis of the tetraspanin complex revealed CDCP1 as a new component of the tetraspanin web, showing that it was co-localized with tetraspanin proteins, including KAI1(CD82), CD81, and CD9 [11]. CDCP1 acts as metastasis enhancer through tyrosine phosphorylation-dependent interactions with Src and PKCδ [12,13]. It is a transmembrane protein with three extracellular CUB domains, named for the first three identified proteins (complement factor C1r/C1s, embryonic sea urchin protein uEGF, and bone morphogenic protein-1) containing such a domain. Highest levels of CDCP1 expression are found in colon, skin, small intestine, and prostate [14-16]. One tetraspanin protein, CD9, has been shown to be a CDCP1-binding protein [12], but certain features of the interaction of CDCP1 with tetraspanin proteins have not been fully defined. Significantly, inhibiting CDCP1 with an anti-CDCP1 antibody was shown to block tumor growth and metastasis in prostate cancer [16].

Recent studies have revealed that KAI1 attenuates EGFR, integrin, and c-Met signaling, confirming the involvement of KAI1 in the intracellular signaling pathways associated with these molecules [3,5]. Moreover, KAI1 expression is reported to inhibit activation of Src kinase in prostate cancer cells [17-20]. The CDCP1-interacting protein Src--the most extensively characterized number of the family of nonreceptor tyrosine kinases--can dominantly contribute to cancer progression, angiogenesis, and metastasis [21]. In particular, Elevation of Src is functionally linked to the development of prostate cancer [22]. Thus, how KAI1 interrupts Src kinase activation--a step required for suppression of metastasis--becomes an essential question.

To better understand how KAI1 is involved in the negative regulation of Src and inhibition of metastasis, we postulated an interrelationship between KAI1 and CDCP1 in regulation of Src activity in TEM. To test this supposition, we here examined the function of KAI1 in CDCP1-mediated Src kinase activation and the consequences of this process, focusing on VEGF expression. Restoration of KAI1 expression in PC3 human prostate carcinoma cells blocked Src activation through negative regulation of CDCP1. Notably, HIF-1α and VEGF expression were significantly inhibited by KAI1-mediated Src inactivation and subsequent VHL up-regulation. Importantly, KAI1 inhibited the expression of CDCP1 and HIF-1α in an *in vivo *tumor xenograft model. These novel findings may indicate that KAI1 functions as a profoundly effective metastasis suppressor in the process of tumor malignancy and angiogenesis through inhibition of CDCP1-mediated Src activation and a subsequent dramatic reduction in HIF-1α and VEGF expression via functional activation of VHL. These studies may shed light on the mechanism by which KAI1 suppresses prostate cancer metastasis.

## Methods

### Cell culture

PC3 cells (ATCC^® ^CRL-1435) were maintained in RPMI medium 1640 supplemented with 10% heat-inactivated fetal bovine serum and antibiotics. Before exposure to hypoxia, culture medium was replaced by a thin layer of fresh medium (0.15 ml/cm^2^). Cells were transferred to a Bactron Anaerobic/Environmental Chamber (Sheldon Manufacturing, Inc.), which was flushed with 1% O_2_, 5% CO_2_, and 95% N_2 _at 37°C. In hypoxia-mimic experiments, cells were incubated with 100 μM of CoCl_2 _under normoxic conditions.

### Plasmids and transfection

Plasmid of GFP tagged KAI1 was gifted by Dr. Kyung Keun Kim (Chonnam National University, Korea). VEGF-luc promoter-reporter construct (2.2 kb) and HA tagged VHL expresson vector were also gifted by Dr. Joohun Ha (Kyunghee University, Korea) and Dr. Hong-Duk Youn (Seoul National University, Korea) respectively. HIF-1α expression plasmid was provided by Addgene. Predesigned small interfering RNA (si-RNA) for KAI1, VHL, and PKCδ were purchased from Santa Cruz Biotechnology, Inc (Santa Cruz, CA, USA). Si-RNA for CDCP1 was purchased from Dharmacon (Lafayette, CO). For transient transfection, PC3 cells were plated on 6-well plate 1 day before transfection as a density of 10^5^/well. After 24 h, cells were transfected with plasmids or siRNA using LipofectAMINE 2000 (Invitrogen, Carlsbad, CA, USA). For stable transfection of KAI1, PC3 (KAI1-/-) cells were transfected with GFP-KAI1 expression plasmid. Vector-transfected cells (PC3-GFP clone #8) and KAI1-expressing PC3 clones (PC3-KAI1 clone #5 and #6) were picked after selection in 800 *μ*g/ml G418.

### Immunoblotting and imuoprecipitation

For polyacrylamide gel electrophoresis (PAGE), the cells were washed with PBS twice and extracted in RIPA buffer (150 mM NaCl, 50 mM Tris, pH 8.0, 1% NP-40, 0.25% sodium deoxycholate, 0.5% sodium lauryl sulfate, 1 mM EDTA). Cell lysates were centrifuged and supernatants were collected. For immunoprecpiation, cell extracts were incubated with antibody (1:1000 dilutions) with constant agitation at 4°C overnight. After electrophoresis, proteins were transferred to nitrocellulose membrane. Membranes were blocked with 1% bovine serum albumin. Then, protein levels were detected using the following commercial antibodies: anti-HIF-1 α, anti-integrin β1, anti-E-cadherin, anti-N-cadherin, and anti-FAK^397 ^(BD Biosciences, Franklin Lakes, NJ, USA); anti-CDCP1, anti-phospho-p130CAS^Y410^, anti-phospho-Src^Y416^, anti-Src, and anti-VHL (Cell Signaling Technology, Danvers, MA, USA); anti-GFP, anti-PKC δ, anti-KAI1, anti-HA, anti-FAK, anti-snail, anti-VHL, anti-paxillin, and anti-β actin (Santa Cruz Biotechnology, Santa Cruz, CA, USA).

### In vitro migration assay

Cell migration assays were performed using Boyden chamber as previously described [18]. Cells were plated on the upper side of a polycarbonate membrane separating two chambers of 6.5 mm Transwell culture plates (Costar, Corning, NY, USA). After 24 hours, cells on the upper face of the membrane were scraped using a cotton swab and cells that had migrated to the lower face of the membrane were stained with DiffQuick (Baxter Scientific, Deerfield, IL USA) Wright-Giemsa Solution.

### In vitro invasion assay

Cell invasion assay was performed on a matrigel invasion chamber (BD Biocoat, BD biosciences, Bedford, MA, USA). This experiment was set up identically to the cell migration assay. After 72 hours, cells remaining upper face of the membrane were scraped using a cotton swab and cells that had invaded to the lower face of the membrane were stained with DiffQuick (Baxter Scientific, Deerfield, IL USA) Wright-Giemsa Solution.

### Reverse Transcription-polymerase Chain Reaction (RT-PCR)

Total RNA was extracted with Trizol reagent (Invitrogen, Carlsbad, CA, USA). The cDNA fragment was amplified by PCR using following specific primers: CDCP1, sense 5'-CTTCAACCTCTCCAACTGTG-3' and antisense 5'-TGGTCTGTGCAGCTTATGGT-3', HIF-1α, sense 5'-CTTGCTCATCAGTTGCCACTT-3', and antisense 5'-GCCATTTCTGTGTGTAAGCAT-3'; VEGF, sense 5'- CGAAGTGGTGAAGTTCATGGATG-3', and antisense 5'- TTCTGTATCAGTCTTTCCTGGTGAG -3'; GLUT1, sense 5'-CGGGCCAAGAGTGTGAA-3', and antisense 5'- TGACGATACCGGAGCCAATG-3', GAPDH, sense 5'-TGCTGAGTATGTCGTGGAGTCTA-3', and antisense 5'-AGTGGGAGTTGCTGTTGAAGTCG-3'; β-actin, sense 5'-GTGGGGGCGCCCAGGCACCA-3', and antisense 5'-CTCCTTAATGTCACGCACCATTTC-3'. PCR was initiated in a thermal cycle programmed at 95°C for 5 min, 94°C for 45 s, 60°C for 2 min, and 72°C for 2 min and amplified for 30 cycles. The amplified products were visualized on 1% agarose gels.

### VEGF- promoter assay

PC3 cells were plated onto 6-well plates (5 × 10^5 ^cells/well), and the following day cells were co-transfected with a VEGF-luc promoter-reporter construct and KAI1 expression vector. Forty-eight hours after transfection, reporter gene activity was measured by assaying luciferase activitiy (Promega, Madison, WI, USA), and normalized to β-galactosidase activities (Promega, Madison, WI, USA).

### Tumor xenograft

Two million PC3 cells were resuspended in 100 μL PBS and injected subcutaneously in the flanks of six-week-old athymic nude mice (Balb/C, Charles River, Japan). Tumor measurements were done with precision calipers and animals were sacrificed after 35 days of injection. Experiments were conducted according to the guidelines for ethical use of animals of our Institution under an approved protocol (approved number of this experiment from ethical committee of animal experiment: KIRAMS 2011-5). Tumors were harvested and fixed overnight in 10% buffered formalin, embedded in paraffin, and sectioned. Primary tumor volumes were calculated with the formula: V = length × (width)^2^/2.

### Immunohistochemistry

Deparaffinized sections were rehydrated and antigen retrieval was performed by 15 minutes incubation in warm trypsin followed by microwave in 10 mM citrate buffer for total of 10. Slides were then blocked with 3% H_2_O_2 _followed by blocking in goat serum and primary incubation at 4°C overnight. The slides were then washed in Tris-NaCl buffer and incubated at room temperature for 60 minutes with anti-CDCP1 antibody (R&D systems, Minneapolis, MN, USA) at a 1:50 dilution, anti-HIF-1 antibody (Thermo Fisher Scientific, MA, USA) at a 1:50 dilution, anti-VHL antibody (Thermo Fisher Scientific, MA, USA) at a 1:50 dilution. Slides were rinsed in Tris-NaCl buffer and incubated for 60 minutes with a biotinylated secondary antibody, biotin polyclonal anti-rabbit immunoglobulin (for CDCP1 and VHL) (BD Transduction Laboratories, Lexington, KY, USA) at a 1:200 dilution, or biotin polyclonal anti-mouse immunoglobulin (for HIF-1) (BD Transduction Laboratories, Lexington, KY, USA) (at a 1:100 dilution. Secondary staining was performed and colorized using Vectastain ABC Kit (Vector Laboratories, Burlingame, CA, USA) and 3,3'-diaminobenzidine (DAB)-H_2_O_2 _substrate (Sigma, St, Louis, MO). Slides were then counterstained with hematoxylin, dehydrated through graded alcohols and xylene and mounted. Slides were studied and imaged under brightfield microscopy.

### Statistical analysis

Data are expressed as means ± standard deviations (SD) of at least three experiments. Statistical significance was determined using Student's t-test for comparisons between two means. The null hypothesis was rejected in cases where *p- *values were < of 0.05. Densitometry of immunoblots was performed by scanning of the exposed film and using The Quantity one 1-D analysis software (Bio-rad Laboratories, Inc, CA, USA)

## Results

### Restoration of KAI1 expression decreases phosphorylation of Src kinase in PC3 prostate carcinoma cells

**Figure 1:**
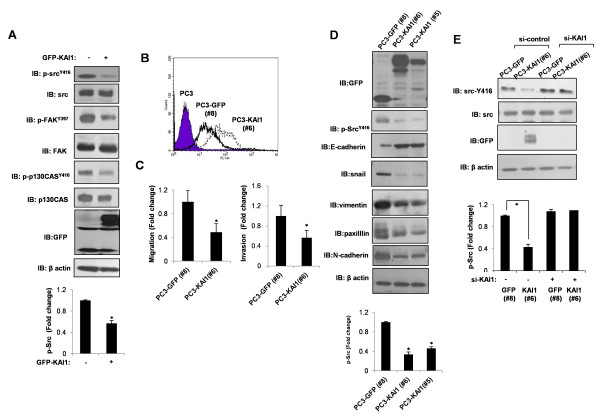
**Phosphorylation of Src at Tyr416 is inhibited by KAI1**. (**A**) After transient transfection of KAI1 in PC3 prostate cancer cells, phospho-Src^Y416^, phospho-FAK^Y397^, and phospho-p130Cas^Y410 ^protein levels were measured by immunoblotting. Densitometric analysis of phospho-Src ^Y416 ^was shown after normalization to beta-actin. (**B**) Vector-transfected cells (PC3-GFP clone #8) and KAI1-expressing PC3 clones (PC3-KAI1 clone #5 and #6) were picked after stable transfection with KAI1 cDNA and selection in 800 *μ*g/ml G418. Cell surface expression of KAI1 was analyzed by flow cytometry. (**C**) The inhibitory effect of KAI1 on migration and invasion was confirmed in KAI1-expressing clones by transwell migration assay and invasion assay, respectively. (**D**) Cell lysates were harvested and immunoblotted for GFP-KAI1, phospho-Src^Y416^, E-cadherin, Snail, vimentin, paxillin, and N-cadherin. Densitometric analysis of phospho-Src ^Y416 ^was shown after normalization to beta-actin. (**E**) Vector-transfected cells (PC3-GFP #8), and KAI1-expressing PC3 clones (PC3-KAI1 #6) were transiently transfected with siRNA against KAI1, and then Src phosphorylation was monitored by immunoblotting. Densitometric analysis of phospho-Src ^Y416 ^was shown after normalization to beta-actin.

It has been proposed that KAI1 inhibits Src activation [17-20]. Because KAI1 was initially identified as a metastasis-suppressor gene in prostate cancer, we investigated the metastasis-suppressor function of KAI1 in PC3 metastatic prostate cancer cells, focusing on Src inhibition. Src, a nonreceptor tyrosine kinase that transduces signals that control metastasis, is autophosphorylated at tyrosine residue 416 upon activation [23]. Thus, we examined the tyrosine phosphorylation of Src. PC3 prostate carcinoma cells, which were previously demonstrated to have lost KAI1 expression, were transiently transfected with a GFP-tagged KAI1 expression vector. As shown in Figure 1A, the level of phosphorylated Src (phospho-Src^416^) was reduced by KAI1 expression. To provide further support for this interpretation, we also monitored the Src substrates, FAK and p130Cas. The level of the tyrosine-phosphorylated, active forms of FAK and p130Cas were decreased by KAI1 expression (Figure 1A). Next, we stably transfected with a GFP-tagged KAI1 expression vector. As PC3 prostate carcinoma cells were devoid of KAI1 expression, Vector-transfected cells (PC3-GFP clone number #8), and KAI1- expressing PC3 clones (clone number #5 and #6) were picked after stable transfection with KAI1 cDNA and selection in 800 *μ*g/ml G418. Cell surface expression of KAI1 was determined by FACS analysis (Figure 1B). Inhibition of migration and invasion in PC3-KAI1 stable transfectants were confirmed by transwell migration assays and invasion assay, respectively (Figure 1C). Figure 1Dshows KAI1 expression in lysates from the PC3-GFP vector clone (#8) and KAI1 stable transfectants (#5 and #6) after immunoblotting with an anti-GFP antibody. The cell motility-inhibitory function of KAI1 was determined by examining the expression of E-cadhesin, Snail, vimentin, paxilllin, and N-cadherin. As shown in Figure 1A, D, KAI1 expression significantly reduced the phosphorylation of Src at Tyr416, suggesting that KAI1 inhibits Src activity. To confirm the effect of KAI1 effect on Src activity, we transfected GFP vector clones (PC3-GFP #8) and KAI1 stable transfectants (PC3-KAI1 #6) with small interfering RNA (siRNA) against KAI1 and analyzed Src phosphorylation. As shown in Figure 1E, knockdown of KAI1 expression in KAI1 stable transfectants (PC3-KAI1#6) reversed the inhibition of Src kinase activity. Therefore, consistent with previous reports, KAI1 expression contributes to Src inhibition and suppression of cell migratory activity in PC3 prostate cancer cells.

### KAI1 acts through negative regulation of CDCP1 to inhibit Src activity

**Figure 2:**
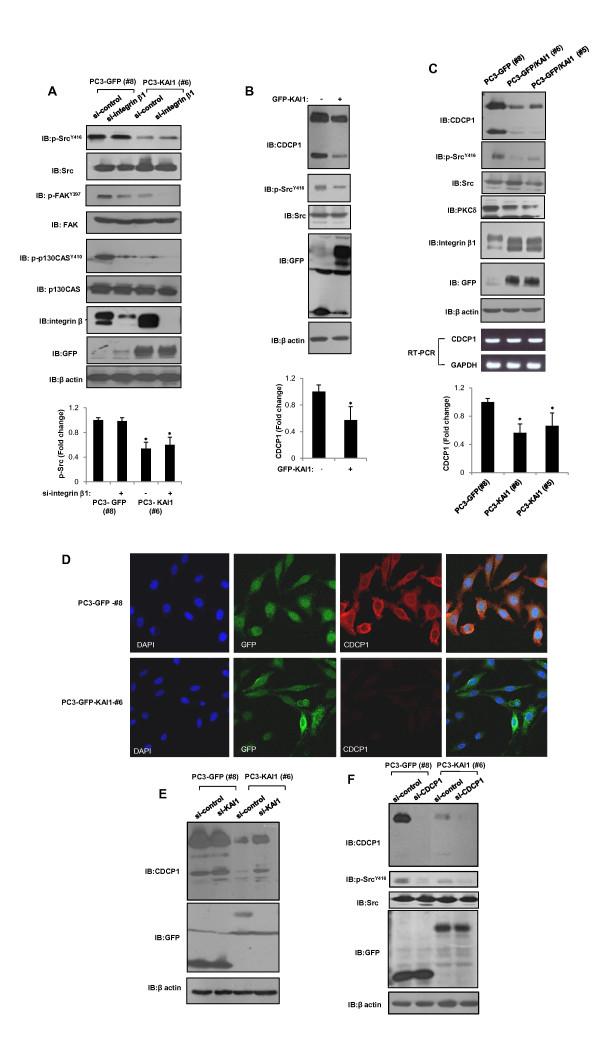
**KAI1-mediated negative regulation of CDCP1 inhibits Src**. (**A**) Vector-transfected cells (PC3-GFP #8) and KAI1-expressing PC3 clones (PC3-KAI1 #6) were transiently transfected with siRNA against integrin β1. Forty-eight hours after transfection, the levels of phospho-Src^Y416^, phospho-FAK^Y397^, phospho-p130Cas^Y410^, and integrin β1 were measured by immunoblotting. Densitometric analysis of phospho-Src ^Y416 ^was shown after normalization to beta-actin. (**B**) PC3 cells were transfected with KAI1. Twenty-four hours after transfection, phospho-Src^Y416 ^and CDCP1 were detected by immunoblotting. Densitometric analysis of CDCP1 was shown after normalization to beta-actin. (**C**) Cell lysates from PC3 vector control (PC3-GFP #8) and KAI1 transfectants (PC3-KAI1 #5 and PC3-KAI1 #6) were analyzed for CDCP1, phospho-Src^Y416^, PKCδ, integrin β1, and GFP-KAI1 protein levels. Densitometric analysis of CDCP1 was shown after normalization to beta-actin. (**D**) CDCP1 proteins were detected by immunofluorescence using anti-CDCP1 antibody and visualized using rhodamine-labeled secondary antibody. (**E**) PC3 cells stably transfected with KAI1 (PC3-KAI1 #6) and vector control (PC3-GFP #8) cells were treated with siRNA against KAI1. After 48 hours, cell lysates were analyzed by immunoblotting to detect CDCP1 and GFP-KAI1. (**F**) After siRNA-mediated knockdown of CDCP1, phospho-Src^Y416 ^levels were monitored by immunoblotting.

Src kinase is a central element in multiple signaling pathways that are important in tumor development, functioning in oncogenesis through its interplay with various upstream and downstream signaling molecules, such as integrins, c-Met, and numerous growth factor receptors [21]. Numerous studies have proposed that KAI1 functions to limit integrin-mediated Src activation [5,18-20]. However, we found that integrin β1 depletion caused no specific changes in Src phosphorylation levels (Figure 2A). Furthermore, altered band shift of integrin beta 1 were observed by KAI1 expression (Figure 2C). Therefore, we investigated another target of KAI1 present in prostate cancer that interferes with Src signaling: CDCP1. It has been noted that a high molecular weight (HMW; 135-140 kDa) form of CDCP1 is processed through interaction with a proteolytic enzyme to a lower molecular weight form (LMW; ~80 kDa) [12,24]. Both HMW and LMW forms of CDCP1 can be tyrosine-phosphorylated by Src kinase. Expression of KAI 1 in PC3 cells significantly reduced the protein levels of both HMW and LMW forms of CDCP1 and its interacting protein, PKCδ (Figure 2B, C). Notably, RT-PCR analyses showed no changes in CDCP1 mRNA levels (Figure 2C). Next, the effect of KAI1 in CDCP1 levels was examined by immunofluorescent microscopy. CDCP1 was clearly stained in PC3 GFP vector clones. By contrast, in the presence of KAI1, loss of CDCP1 were observed in PC3-KAI1 stable transfectant (Figure 2D). To confirm KAI1-mediated negative regulation of CDCP1, we treated siRNA of KAI1 in GFP vector clones (#8) and KAI1 stable transfectants (#6). SiRNA-mediated knockdown of KAI1 reversed the decrease in CDCP1 protein (Figure 2E). To clarify the effect of KAI1 on CDCP1-mediated Src activation, we treated GFP vector clones (#8) and KAI1 stable transfectants (#6) with siRNA against CDCP1 and examined Src phosphorylation. As shown in Figure 2F, siRNA-mediated knockdown of CDCP1 resulted in suppression of Src phosphorylation, an effect that was also pronounced in KAI1 stable transfectants (#6). Hence, KAI1 acts through down-regulation of CDCP1 protein to inhibit Src activation in PC3 prostate cancer cells.

### KAI1 increases VHL protein levels

**Figure 3:**
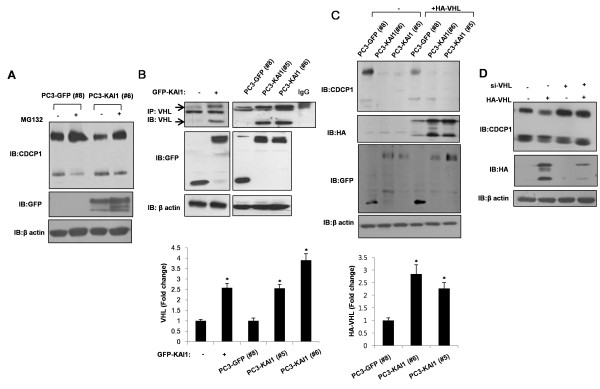
**KAI1 increases VHL protein levels**. (**A**) PC3 cells stably transfected with KAI1 (PC3-KAI1 #6) or vector control (PC3-GFP #8) cells were treated with MG132. After 12 hours of treatment, cell lysates were prepared and the levels of CDCP1 were measured by immunoblotting. (**B**) Cell lysates were prepared from PC3 cells transiently transfected with KAI1 and from stable transfectants. VHL expression was analyzed by immunoprecipitation. Densitometric analysis of VHL was shown after normalization to beta-actin. (**C**) PC3 cells stably transfected with KAI1 (PC3-KAI1 #5 and PC3-KAI1 #6) or vector control (PC3-GFP #8) were transiently transfected with HA-tagged VHL. Twenty-four hours after transfection, cells were harvested, and the levels of CDCP1 and VHL protein were analyzed by immunoblotting. Densitometric analysis of HA-VHL was shown after normalization to beta-actin. (**D**) PC3 cells were transfected with HA-tagged VHL, and then treated with siRNA against VHL. The protein levels of CDCP1 and HA-VHL were assessed by immunoblotting.

A recent report has linked loss of the VHL protein to up-regulation of CDCP1 through HIF-1α-dependent transcriptional activation [25]. The fact that KAI1 reduced CDCP1 protein levels in PC3 cells without significant altering CDCP1 mRNA levels (see Figure 2C) suggested that a posttranscriptional mechanism was responsible for this action. To test this, we examined the involvement of proteasome-dependent degradation of CDCP1 in KAI1-expressing PC3 cells. As shown in Figure 3A, treatment with the proteasome inhibitor MG132 blocked the KAI1-induced reduction in CDCP1. Earlier reports have proposed that the tumor-suppressor function of the VHL protein reflects the role of VHL as an ubiquitin ligase of the transcription factor HIF-1α, which is known as a master regulator of hypoxic response. Moreover, it has been shown that Src destabilizes the VHL protein [26]. Collectively, these observations suggest the possibility that KAI1, acting through inhibition of Src, stabilizes VHL, resulting in the ubiquitin/proteasome-dependent degradation of various proteins. Thus, we hypothesized that KAI1 could inhibits CDCP1-mediated enhancement of Src activation, resulting in subsequent stabilization of VHL. Although the basal level of VHL could not be clearly detected, VHL protein was readily observed in KAI1-expressing PC3 cells, indicating that the restoration of KAI1 increased VHL protein levels (Figure 3B). In order to confirm the increase in VHL protein, we transfected GFP vector clones (PC3-GFP#8) and KAI1 stable transfectants (PC3-KAI1 #5 and PC3-KAI1 #6) with an HA-tagged VHL expression vector and then analyzed the amount of VHL protein. As shown in Figure 3C, there was a dramatic increase in VHL protein levels in KAI1 stable transfectants (PC3-KAI1#5 and PC3-KAI1 #6) transiently overexpressing VHL, confirming that KAI1 increased VHL protein level. Notably, overexpression of VHL in PC3 cells was associated with a decrease in CDCP1 protein levels (Figure 3D). The VHL-induced decrease of CDCP1 is consistent with the recovery of CDCP1 levels by treatment of KAI1 stable transfectants (PC3-KAI1 #6) with MG132 (Figure 3A). Taken together, these results suggest that increased VHL activity in the context of KAI1 protein expression destabilizes the CDCP1 protein, implying that KAI1 increases VHL protein levels and reduces the levels of CDCP1.

### KAI1 inhibits the induction of HIF-1α

**Figure 4:**
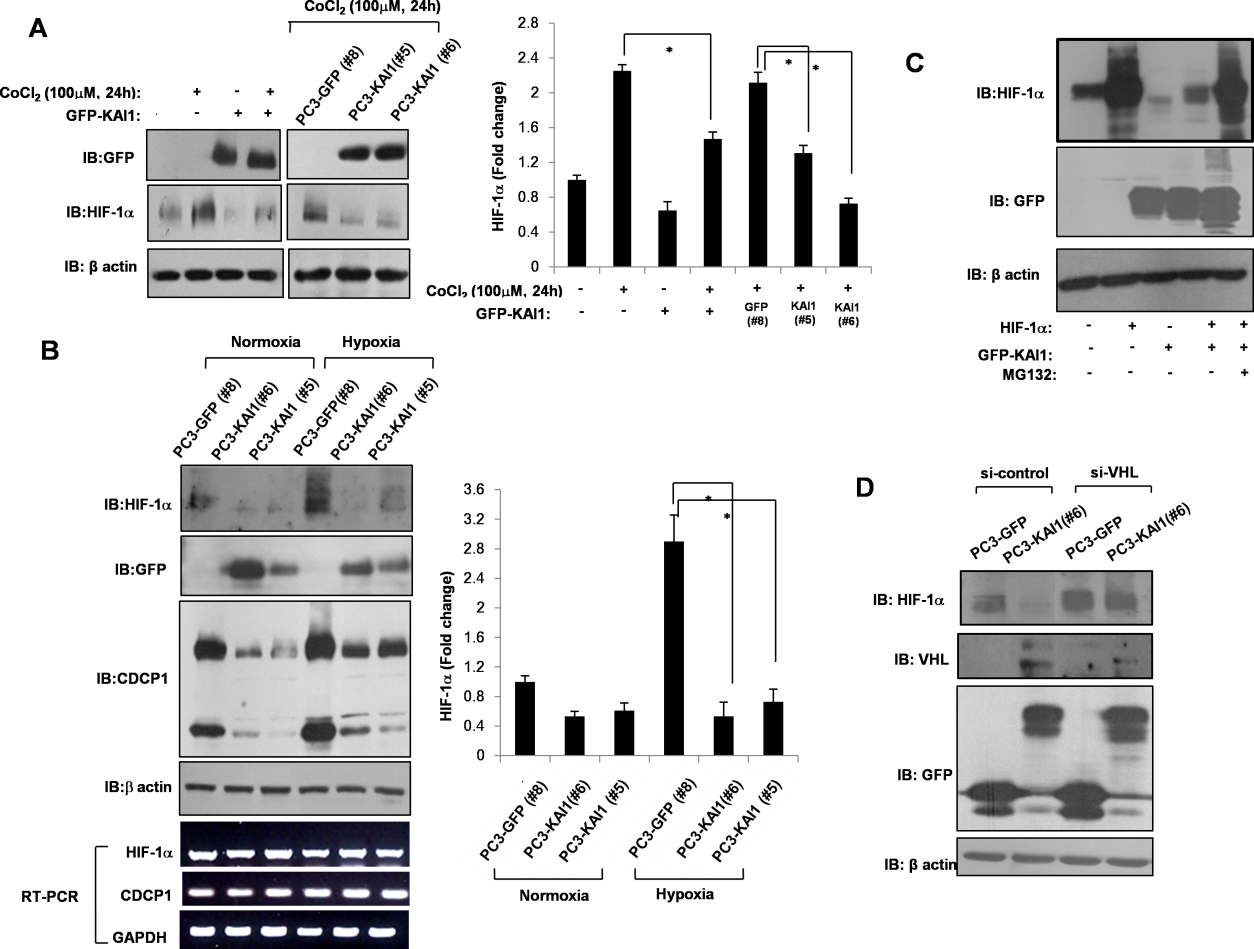
**KAI1 blocks HIF-1α induction**. (**A**) After transfection of KAI1, cells were treated with 100 μM CoCl_2 _for 24 hours. PC3 vector control (PC3-GFP #8) and KAI1 transfectants (PC3-KAI1 #5 and PC3-KAI1 #6) were also treated with 100 μM of CoCl_2 _for 24 hours. Cells were harvested and immunoblotted for detection of HIF-1α and GFP-KAI1. Densitometric analysis of HIF-1α was shown after normalization to beta-actin. (**B**) PC3 vector control (PC3-GFP #8) and KAI1 transfectants (PC3-KAI1 #5 and PC3-KAI1 #6) were exposed to hypoxia for 24 hours. The levels of HIF-1α, CDCP1, and GFP-KAI1 proteins were assessed by immunoblotting. Densitometric analysis of HIF-1α was shown after normalization to beta-actin. HIF-1α and CDCP1 mRNA levels were measured by RT-PCR. (**C**) PC3 cells were cotransfected with KAI1 and HIF-1α, then treated with the proteasome inhibitor, MG132. HIF-1α levels were assessed by immunoblotting. (**D**) Stable KAI1-expressing (PC3-KAI1 #6) and vector control (PC3-GFP #8) PC3 cell clones were treated with siRNA against CDCP1 and VHL. Forty-eight hours after transfection, HIF-1α, CDCP1, and VHL protein levels were measured by immunoblotting.

Increased levels of Src kinase have been reported to increase VEGF production in angiogenesis [21]. VEGF expression, in turn, is normally up-regulated by HIF-1α in response to decreases in oxygen concentration; in normoxia, VHL proteins ubiquitinate and promote the proteasome-dependent degradation of HIF-1α, thereby keeping HIF-1α levels and VEGF expression low [8]. Collectively, these observations form the basis for the important idea that disruption of Src signaling leads to suppression of cancer progression and angiogenesis. To determine whether the inhibition of CDCP1-enhanced Src activation by KAI1 exerts an anti-angiogenic action, we examined the effect of KAI1 on HIF-1α, which functions as a master regulator of VEGF expression. As shown in Figure 4A, treatment with CoCl_2 _(100 μM), which mimics hypoxia, increased HIF-1α levels in PC3 cells, an effect that was dramatically inhibited by transient or stable expression of KAI1. Next, GFP vector clones (PC3-GFP#8) and KAI1 stable transfectants (PC3-KAI1 #5 andPC3-KAI1 #6) were exposed to hypoxia (1% O_2_) for 24 hours, then the effects of KAI1 on hypoxia-induced HIF-1α up-regulation were determined. Similar to the effects observed with CoCl_2_, HIF-1α protein levels under hypoxic conditions were significantly decreased by KAI1 expression (Figure 3B). There were no changes in HIF-1α mRNA expression, suggesting that these effects of KAI1 expression were posttranslational (Figure 3B). Interestingly, the basal levels of HIF-1α were also diminished by KAI1 expression in normoxic conditions (Figure 3B). As noted previously, hypoxia did not alter HIF-1α mRNA expression [8]. Hypoxia up-regulated the CDCP1 protein, also without changing CDCP1 mRNA levels (Figure 4B). Although CDCP1 was increased by hypoxia, it was still repressed by KAI1 (Figure 4B). Collectively, these results suggest that KAI1 is involved in the posttranscriptional regulation of HIF-1α. Because it is known that HIF-1α is rapidly degraded under normoxia by the ubiquitin-proteasome pathway, we assessed the involvement of proteasomal degradation of HIF-1α in the KAI1-mediated inhibition of HIF-1α induction. After cotransfection of HIF-1α and KAI1, PC3 cells were treated with the proteasome inhibitor MG132. KAI1 decreased the levels of HIF-1α protein, an effect that was prevented by pretreatment with MG132 (Figure 4C). We also found that depletion of VHL protein resulted in the recovery of HIF-1α protein levels in KAI1 stable transfectants (Figure 4D), consistent with the purported role of VHL protein as a ubiquitin ligase for HIF-1α in the proteasome degradation pathway. Thus, the CDCP1-induced enhancement of Src activation may be deranged by the expression of KAI1, leading to the subsequent abrogation of HIF-1α induction.

### KAI1 inhibits VEGF expression

**Figure 5:**
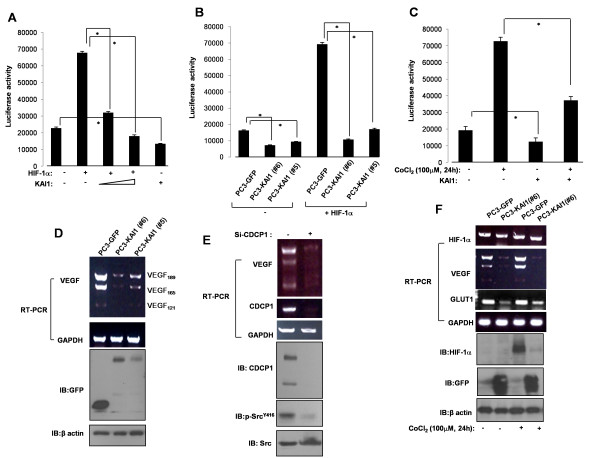
**KAI1 decreases VEGF expression**. (**A**) PC3 cells were cotransfected with a VEGF promoter (2.2 kb)-luciferase reporter plasmid and HIF-1α and GFP-KAI1 expression plasmids. Forty-eight hours after transfection, cell lysates were analyzed for luciferase expression. (**B**) PC3 cells stably transfected with KAI1 (PC3-KAI1 #5 and PC3-KAI1 #6) or vector control (PC3-GFP #8) were cotransfected with a VEGF promoter-luciferase reporter plasmid and a HIF-1α expression vector. Forty-eight hours after transfection, cell lysates were analyzed for luciferase activity. (**C**) PC3 cells were cotransfected with a VEGF promoter-luciferase reporter plasmid and a GFP-KAI1 expression plasmid. Twenty-four hours after transfection, cells were treated with 100 μM CoCl_2 _for 24 hours, and then luciferase activity was measured. (**D**) Total RNA from PC3 cells stably transfected with KAI1 (PC3-KAI1 #5 and PC3-KAI1 #6) or vector control (PC3-GFP #8) was isolated, and VEGF mRNA expression was analyzed by RT-PCR. (**E**) After treatment of si-CDCP1, PC3 cells were harvested and VEGF mRNA expression was analyzed by RT-PCR. (**F**). PC3 cells stably transfected with KAI1 (#6) or vector control (PC3-GFP #8) were exposed to CoCl_2 _(100 μM) for 24 hours. Total RNA was extracted and mRNA levels were quantified by RT-PCR.

Having demonstrated that KAI1 induces the degradation of HIF-1α, we sought to determine the effect of KAI1 expression on VEGF promoter activity. For these experiments, we transiently transfected PC3 cells with a luciferase reporter plasmid containing a 2.2-kb region of the VEGF promoter and measured luciferase activity. As expected, transfection of PC3 cells with HIF-1α induced an increase in VEGF promoter activity; notably, cotransfection of KAI1 inhibited HIF-1α-induced promoter activity (Figure 5A). Interestingly, KAI1 expression alone decreased VEGF promoter activity (Figure 5A, C). We also tested VEGF promoter activity in KAI1 stable transfectants, cotransfecting PC3-vector clone (PC3-GFP #8) and PC3-KAI1 stable clones (PC3-KAI1 #5 andPC3-KAI1 #6) with an HIF-1α expression vector and the VEGF reporter plasmid. Similar to the results shown in Figure 5A, VEGF promoter activity was diminished in KAI1-expressing cell lines (Figure 5B). Moreover, CoCl_2 _(hypoxia mimic) induced an increase in VEGF promoter activity that was reduced by KAI1 expression (Figure 5C). These data support the hypothesis that KAI1 induces HIF-1α degradation and subsequent transcriptional inactivation of VEGF through the induction of VHL. RT-PCR analyses, which confirmed that PC3 prostate cancer cells express high levels of VEGF, showed that KAI1 expression repressed the expression of three VEGF isoforms (VEGF_189_, VEGF_165_, and VEGF_121_; Figure 5D). Since KAI1 negatively regulated CDCP1, we treated siRNA of CDCP1 and analyzed the VEGF expression. Knockdown of CDCP1 decreased the VEGF level (Figure 5E). In addition, the inhibitory effect of KAI1 on VEGF expression was also observed in the context of CoCl_2_-induced up-regulation of VEGF (Figure 5F). Glucose transporter-1 (GLUT1), another transcriptional target of HIF-1α, was also inhibited by KAI1 expression. Thus, the KAI1-induced decrease in HIF-1α by KAI1 manifests as transcriptional inhibition of VEGF.

### KAI1 inhibits HIF-1α and CDCP1 expression in tumor xenografts

**Figure 6:**
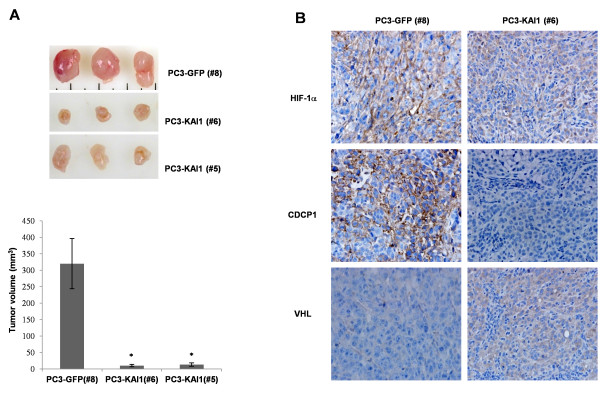
**KAI1 inhibits HIF-1α and CDCP1 expression and VEGF secretion in tumor xenografts**. (**A**) PC3 cells stably transfected with KAI1 (PC3-KAI1 #5 andPC3-KAI1 #6) or vector control (PC3-GFP #8) were resuspended in PBS (2 × 10^6 ^cells/100 μl) and injected subcutaneously into the flanks of athymic nude mice. Animals were sacrificed 35 days after injection, at which time tumors were collected and photographed. Tumor volumes were measured using precision calipers (**B**) Photomicrographs showing expression of HIF-1α, CDCP1, and VHL protein in tumor sections by immunohistochemistry.

KAI1 is known to inhibit metastasis without affecting primary tumorigenesis [5]. However, previous studies have demonstrated that over-expression of KAI1 can lead to significant cell death, described as apoptotic, or autophagic, and caspase-independent [27-29]. In addition, we observed that KAI1 expression was associated with dramatic down-regulation of HIF-1α and VEGF, both of which are known to be essential for survival under conditions of hypoxic stress. Hence, KAI1-expressing cancer cells would be expected to exhibit growth retardation or cell death. To test this, we examined the effect of KAI1 on primary tumor formation and HIF-1α and VEGF expression in a tumor xenograft model. The flanks of nude mice were subcutaneously injected with PC3-vector clone (PC3-GFP #8) or PC3-KAI1 stable clones (PC3-KAI1 #5 andPC3-KAI1 #6). Five weeks after injection, mice were sacrificed and tumor volumes were measured. As shown in Figure 6A, the volume of tumors was significantly reduced in mice injected with KAI1 stable transfectants (PC3-KAI1 #5 and PC3-KAI1#6) compared with those injected with PC3 vector cell lines (PC3-GFP #8). These data suggest that KAI1 expression disrupted the ability of cancer cells in solid tumors to acquire the metastatic potential necessary to overcome hypoxic stress. Next, we performed immunohistochemistry to detect HIF-1α, CDCP1 and VHL protein in tumor tissue. The analyses showed that CDCP1 proteins were barely detectable in tumors derived from KAI1-expressing cell lines; in contrast, VHL immunoreactivity coincided with KAI1 expression. With our expectation, HIF-1α level in KAI1 expressing tumor was significantly low as compared with control (Figure 6B). These results correlate well with our protein expression data obtained from immunoblot analyses. Collectively, these observations demonstrate that the metastasis-suppressive activity of KAI1 is highly relevant to the down-regulation of CDCP1 and suppression of HIF-1α expression in prostate cancer.

## Discussion

The purpose of this study was to establish the precise mechanism by which KAI1 (CD82), a metastasis-suppressor gene initially discovered in prostate cancer, inhibits Src and affects the metastasis of prostate carcinoma. Clinical cancer studies have linked a high level of KAI1 expression with a good prognosis. Conversely, the loss of KAI1 expression is correlated with metastasis in tumor progression [3,5]. However, how KAI1 functions to suppress metastasis has not yet been clearly established. Tetraspanins, including KAI1, may play a role in the plasma membrane as organizers of multi-molecular complexes that contain not only tetraspanins but also numerous cell membrane components. The TEM has been suggested to serve as a signal-networking platform that shifts the characteristics of a cancer between primary tumor and a metastatic phenotype [30,31]. Thus, it is of special interest to define the constituents of this special domain and their interrelationships.

Current studies have proposed that KAI1 functions to inhibit Src kinase activity, but the identified mechanisms have been limited to associations with integrins, EGFR, and c-Met [5]. It has been proposed that KAI1 attenuates EGFR signaling and inhibits cell motility in breast cancer [32]. However, other study reported no alteration of EGFR signaling in response to KAI1 expression in DU145 and PC3 prostate cancer cell lines [18]. It is generally accepted that c-Met can play a role in the metastasis-suppression activity of KAI1 in prostate cancer [33,34]; however, inhibition of this pathway and Src kinase by KAI1 expression appear to be independent of one another [18]. Similarly, and consistent with a previous report [35], we found no connection between integrin complexes and Src inhibition, showing that depletion of integrin β1, the major component of integrin complexes, had no specific effect on Src phosphorylation status but decrease of phosphorylated FAK and p130CAS, downstream effector of integrin complexes, in PC3 cells (Figure 2A). Therefore, the absence of a connection between these KAI1 targets and inhibition of Src kinase activity prompted us to investigate another target of KAI1.

Together with KAI1, CDCP1 has been reported to be a component of the TEM in colon cancer, but its function in this context has remained poorly characterized [11]. CDCP1 is a transmembrane protein that primarily associates with Src and PKCδ in a phosphorylation-dependent manner and may act as a scaffold for various interacting proteins in the plasma membrane [12]. Generally, CDCP1 is highly phosphorylated and functionally activated in metastatic cancer; dysregulated expression of CDCP1 is associated with tumor malignancy [14,36-38]. Tyrosine phosphorylated CDCP1 has been linked to cell detachment from the extracellular matrix and subsequent cell migration [39]. The kinase activity of Src is essential for the initial CDCP1 phosphorylation; Src-activated CDCP1, in turn, further potentiates Src kinase activity [25,40], although the mechanism for this latter effect is not clear. Our data showed that KAI1-induced inhibition of CDCP1 protein resulted in an almost complete elimination of Src phosphorylation (Figure 2F). This observation suggests that the function of CDCP1 protein, which acts as a positive regulator of Src kinase, may be lost due to KAI1 expression in prostate cancer, raising the question of the possible existence of a feedback control mechanism between CDCP1 and Src.

CDCP1 gene regulation has been investigated in various cancer cell lines. PC3 cells show abundant expression of CDCP1 protein and a low frequency of methylation in transcription-initiation sites [41]. We found that both HMW and LMW forms of CDCP1 are expressed in PC3 cells (Figure 2). Although the precise mechanism by which CDCP1 is cleaved has not been defined, proteolytic processing by a serine protease results in the generation of the LMW form of CDCP1 and a subsequent increase in LMW phosphorylation [24]. Our data clearly showed that KAI1 expression decreased the level of both HMW and LMW forms without altering CDCP1 mRNA levels (Figure 2), suggesting that the KAI 1-induced decrease in CDCP1 in PC3 cells reflects the operation of a posttranscriptional mechanism. To test this, we initially treated PC3 cells with cytochalasin D, which blocks cytoskeletal movement, with the goal of determining whether KAI1 affected the endocytic trafficking of CDCP1. We found no effect of this actin-depolymerizing reagent on the KAI1-induced decrease in CDCP1 (data not shown). As demonstrated in previously reported proteomic analyses, CDCP1 and KAI1 are colocalized with various proteases in the TEM [11], raising the possible involvement of KAI1 in protease activity. Since serine protease-induced proteolysis of CDCP1 has been documented [24], we treated PC3 cells with protease inhibitors and monitored the levels of CDCP1 under conditions of KAI1 over-expression, but found no significant change in either HMW or LMW forms of CDCP1 (data not shown). Finally, to test the involvement of proteasome-dependent degradation in the process of CDCP1 down-regulation, we treated PC3 cells with the proteasome inhibitor, MG132, and found that inhibiting the proteasome prevented the KAI1-dependent reduction in CDCP1 levels (Figure 3A). Because CDCP1 is reported to localize in the TEM of colon cancer by proteomic analysis, we could not exclude a possible interaction between KAI1 and CDCP1. In experiments designed to determine whether these two membrane proteins were co-localized in PC3 cells, it was hard to detect an interaction of CDCP1 with KAI1 due to KAI1-induced loss of CDCP1 protein level. Our observations indicate that KAI1-induced down-regulation of CDCP1 reflects effects on CDCP1 protein stability, but unknown mechanisms may also participate in this phenomenon.

Angiogenesis is an indispensible step in the progression of prostate cancer, and VEGF has emerged as a critical proangiogenic growth factor in prostate carcinogenesis [22,42,43]. Hypoxia is the environmental factor best known for its ability to induce cancer metastasis; it also stabilizes HIF-1α and up-regulates the expression of VEGF, which, in turn, induces the formation of tumor-feeding vessels [6]. Moreover, it is known that Src kinase not only affects cell proliferation and migration, it also control angiogenesis via up-regulation of VEGF expression [44]. However, few studies have focused on the role of KAI1 activity in VEGF expression and vasculature formation in tumors. Thus, given the critical role of KAI1 in inhibiting Src kinase and our motivation to identify a plausible mechanism to account for KAI1 effects in carcinogenesis, we examined HIF-1α and VEGF expression in the context of KAI1 expression. We found that HIF-1α and VEGF expression were dramatically inhibited upon restoration of KAI1 expression (Figures 4 and 5). These findings were supported by the results obtained using an *in vivo *xenograft model, which showed that KAI1-expressing tumor volumes were significantly reduced compared with those of controls (Figure 6). Moreover, whereas CDCP1 and HIF-1α expression were reduced in KAI1-expressing tumor tissue, VHL expression was clearly augmented. Thus, our data provide a mechanistic basis for the clear correlation between the loss of KAI1 in cancer and poor prognosis. It has recently been demonstrated that Src can promote oncogenesis through destabilization of the VHL tumor suppressor [26]. Importantly, functional inactivation of VHL, including through germline mutations, has been well documented in highly vascularized tumors such as renal cell carcinomas, hemangiosarcomas, and pheochromocytomas [9]. The tumor-suppressive function of VHL is best viewed in the context of its role as an E3 ubiquitin ligase that targets various substrates, including HIF-1α and atypical PKC [8]. Two forms of VHL, approximately 30 and 18 kDa, exist, but the significance of the two forms is unknown [45]. It has been suggested that VHL is extremely unstable when not complexed with components of ubiquitin ligase complexes, such as elongin B and elongin C [9]. This observation may account for the difficulty in detecting VHL protein expression and may help to explain why most VHL functions have been examined by overexpressing or immunoprecipitating VHL. We observed that VHL protein levels were increased by KAI1 expression (Figure 3B, C). How KAI1 negatively regulates CDCP1 remains uncertain, but our data clearly suggest that this effect of KAI1 involves the regulation of CDCP1 protein stability. Intriguingly, VHL over-expression decreased CDCP1 levels; this result was confirmed by treating cells with siRNA against VHL, which had the opposite effect on CDCP1 levels (Figure 3D). VHL protein is expressed in various normal and cancer tissues, where it is localized in the cytosol or membrane [46]. In human renal cell carcinoma, its presence in the membrane is significantly associated with a missense mutation [10,47]. These reports prompted us to further consider changes in subcellular localization of VHL in the process of tumorigenesis. In addition, we observed the existence of VHL in both of cytosol and membrane fractions in VHL overexpressing PC3 cells (data not shown). This observation raises the possibility of recruitment of VHL from cytosol to plasma membrane and ubiquitination of unknown substrates located in the membrane. Although we could not observe co-localization of CDCP1, VHL and KAI1 in cell membrane or TEM, these proteins would be expected to coexist in the plasma membrane during the process of prostate carcinogenesis.

## Conclusion

We report here that KAI1 acts in the process of tumor malignancy and angiogenesis by profoundly suppressing metastasis via inhibition of CDCP1-enhanced Src activation and functional activation of VHL. This KAI1-induced VHL activation causes HIF-1α degradation and, ultimately, down-regulation of VEGF expression. These results provide insight into how KAI1 suppresses prostate cancer metastasis. Although we focused our efforts here on CDCP1-Src inhibition by KAI1 and their effects on VEGF expression, KAI1 expression may also influence other key processes in prostate carcinogenesis.

## Abbreviations

CDCP1: CUB-domain-containing protein 1; HIF-1α: Hypoxia inducible factor 1 α; VHL: Von Hippel-Lindau; VEGF: Vascular Endothelial Growth Factor; TEM: Tetraspanin-Enriched Microdomain; GAPDH: Glyceraldehydes-3-phosphate dehydrogenase; GLUT1: Glucose Transporter 1; siRNA: small interfering RNA

## Competing interests

The authors declare that they have no competing interests.

## Authors' contributions

JJP performed experiments; YBJ did tumor xenograft and immunohistochemistry; YJL helped performing experiments. JSL helped designing the research; YSL helped designing the research; YGK designed, analyzed data, and wrote paper; ML designed, analyzed data, and wrote paper. All authors have read and approved this manuscript

## Pre-publication history

The pre-publication history for this paper can be accessed here:

http://www.biomedcentral.com/1471-2407/12/81/prepub
